# Supplementary material for: One-Step In Situ Patternable Reduction of a Ag–rGO Hybrid Using Temporally Shaped Femtosecond Pulses
Source: Materials (Basel). 2022 Jan 12;15(2):563. doi: 10.3390/ma15020563 (PMC8781587; doi:10.3390/ma15020563)
Supplement: Supplementary file 1 [file materials-15-00563-s001.zip › materials-1533044-supplementary.pdf]

Article

One–Step In-Situ Patternable Reduction of Ag–rGO Hybrid using Temporally-Shaped Femtosecond Pulses

Quan Hong,[a] Lan Jiang,[*a,b] Sumei Wang,[a,b] Ji Huang,[a] Jiaxin Sun,[a] Xin Li,[a,b] Pei Zuo,[a,b] Jiangang Yin [c] and Jiangang Lu [c]

[a] Laser Micro/Nano-Fabrication Laboratory, School of Mechanical Engineering, Beijing Institute of Technology, 5 South Zhongguancun Street, Haidian District, Beijing 100081, P.R. China.
[b] Beijing Institute of Technology Chongqing Innovation Center, Chongqing, 401120, China.
[c] Han's Laser Technology Industry Group Co., Ltd. 6 building WanYan Industry Zone, Haoye Road, Fuyong Town, Baoan District, Shenzhen City, Guangdong Province, P.R. China.
*Corresponding author.
Email address: @bit.edu.cn

1. Experimental setup

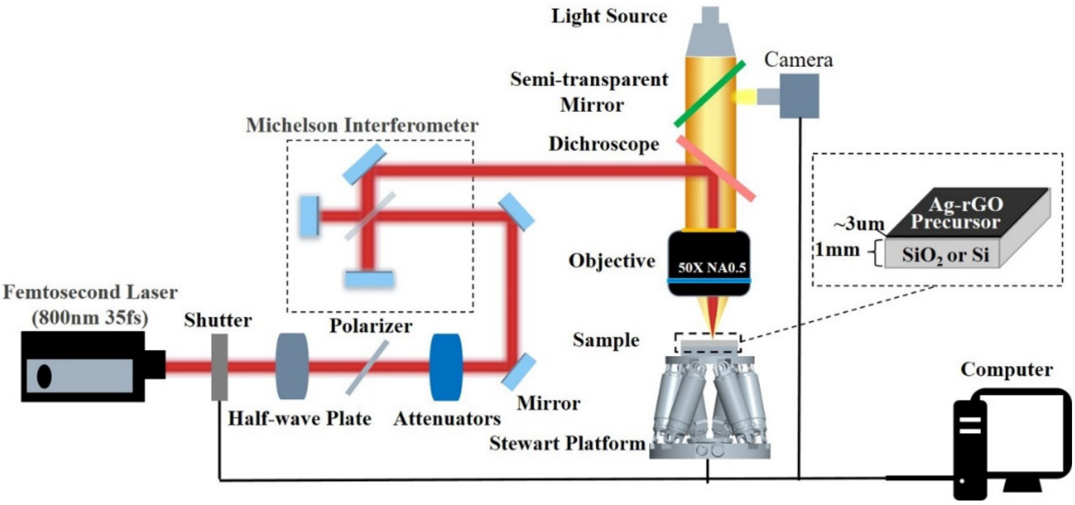

Figure S1. Experimental setup.

2. Controllable Deposition of the Ag–rGO Hybrid

The rectangular patterns were formed through line-by-line scanning with a line interval of 1 μm, as displayed in Figure. S2(a). When the fluence increased to 1.288 J/cm^2, the substrate was over-patterned, resulting in a failure to form a precise rectangular pattern, as depicted in Figure S2(c). These patterns are assumed to be the repetitive deposition of recast layers during ablation. When patterning along the scanning direction shown in the upper corner of Figure 1(a), the recasting zone on the left was continuously processed while it accumulated in the opposite direction, which resulted in inhomogeneous deposition on the left edge of the pattern, as depicted in Figure S2(b). Although inhomogeneous deposition was present at the left edge due to differing amounts of overlapped pulses, the remainder of the pattern exhibited favorable homogeneous properties. At relatively low energy, the clear boundary of the pattern indicated that the microlandscaping process had an acceptable resolution (Figure. S2(d)).

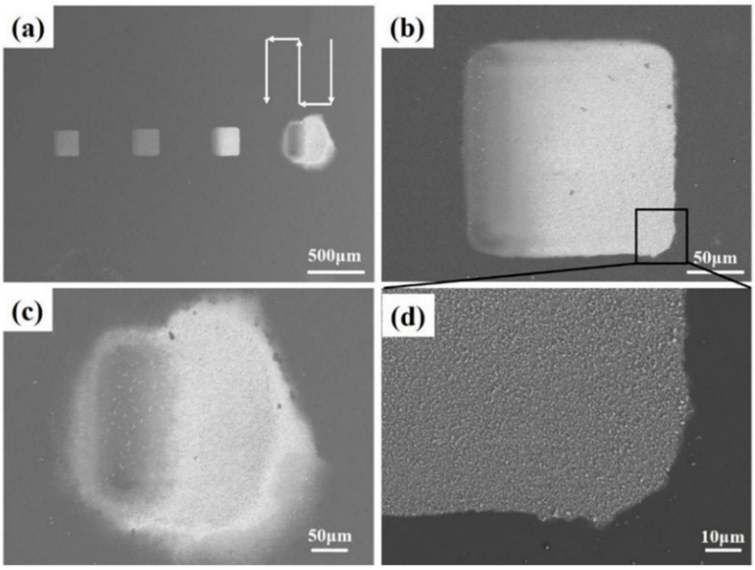

Figure S2. Morphology of the Ag–rGO hybrid on SiO_2 treated with the femtosecond laser at various fluences: (a) rectangular patterns with different fluences (from left to right, the laser power was as follows: 0.178, 0.354, 0.708, and 1.288 J/cm^2), (b, c) detailed views of the third and the last rectangular patterns, and (d) boundary of the third rectangular pattern.

3. TGA Characterization

To satisfy the mass requirement for the TG testing, thick film prepared by simple vacuum filtration were used. The GO dispersion and the mixed solution of GO and $AgNO_3$ were filtered through the membranes (25 nm pore size) to form the restacked thick film. The prepared films were then cut into pieces with its mass in the order of 10mg for TG testing. Corresponding TG curves can be seen in Figure S3. As we can see, significant mass loses of 12% and 6% were observed in the range up to 100℃ for GO and $AgNO_3$-GO respectively. A more dramatic mass loss measured in the range of 100-300℃, with the losses measured being 28.2% and 25.3% for GO and $AgNO_3$-GO, respectively. It is reported that both GO and Ag-GO nanocomposite suffer similar weight loss bellow 100 ℃ which can be attributed to the removal of absorbed water. The doping of silver nanoparticles can reduce unstable oxygenated functional groups on the AgGO sample leading to lower rate of weight loss when compared with that of GO[1, 2]. Considering the hydrophilic nature of GO and the non-vacuum fabrication environment, we considered the water is absorbed on the surface of the Ag-rGO precursor.

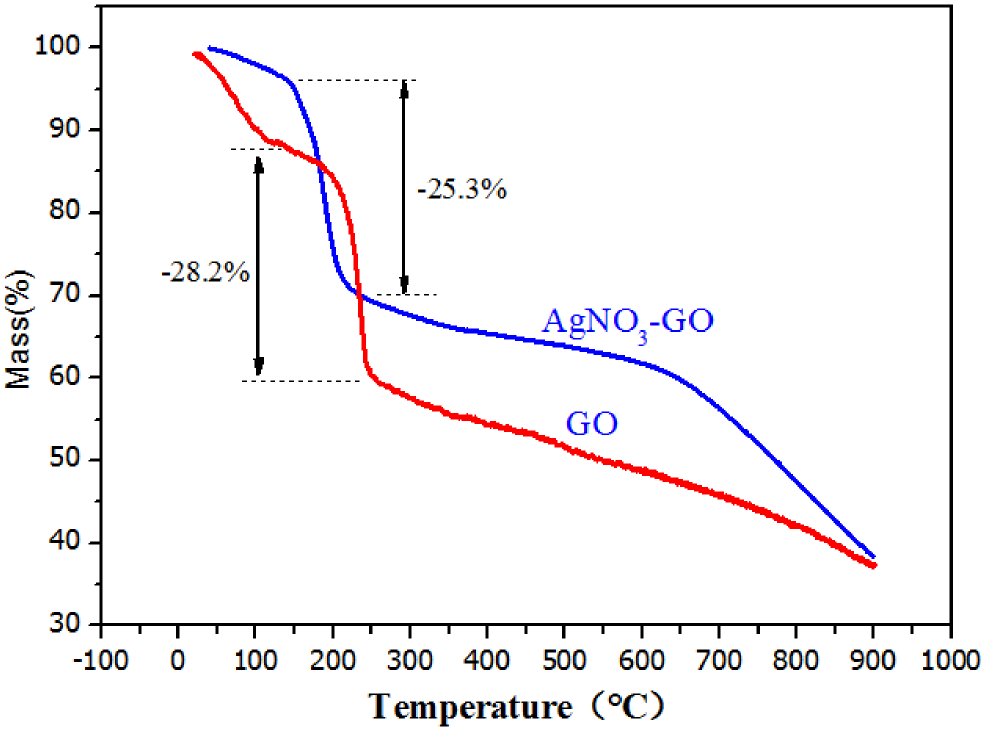

Figure S3. TGA curves (under N2 flow) for the GO and $AgNO_3$-GO.EDS results and morphology of the Ag–rGO hybrid: (a) morphology of the coralloid-like Ag–rGO hybrid when the fluence was 0.035 J/cm^2, (b) EDS results for the zone indicated by arrow 2, and (c) EDS results for the zone indicated by arrow 1.

4. Mapping the Ag–rGO Hybrid on Different Substrates Under Different Fabrication Conditions

4.1. Preparation of the Ag–rGO Precursor Displayed in Figure S4

Silver nitrate solution (5 mM) was prepared by adding 4.25 mg of silver nitrate powder into 5 mL of deionized (DI) water and sodium citrate solution, which was formed by adding 0.294 g of sodium citrate powder into 10 mL of deionized water.

A total of 10 mL of GO dispersion (0.5 mg/mL) was mixed with silver nitrate solution under sufficient stirring. Several drops of the mixed solution were homogeneously dispersed on the Si substrate through spin-coating. The substrate was then dried in a vacuum drying oven at 50°C overnight to form the Ag–rGO precursor.

A total of 10 mL of GO dispersion (0.5 mg/mL) was mixed with silver nitrate solution under sufficient stirring. Several drops of the mixed solution were homogeneously dispersed on the SiO_2 substrate through spin-coating. The substrate was then dried in a vacuum drying oven at 50°C overnight to form the Ag–rGO precursor.

Several drops of the GO dispersion (0.5 mg/mL) were homogeneously dispersed on the SiO_2 substrate through spin-coating. The substrate was then dried in a vacuum drying oven at 50°C overnight to form the Ag–rGO precursor.

4.2. Mapping the Ag–rGO Displayed in Fig S4

The silicon substrate coated with the corresponding Ag–rGO precursor layer (Table S1) was mounted on the six-axis Stewart platform, and the Ag–rGO precursor layer was ablated by focusing a femtosecond laser on the substrate surface. The substrate was then washed with DI water several times until the entire laser-untreated area was cleaned thoroughly. The residual pattern was dried under ambient conditions.

The silica substrate coated with the corresponding Ag–rGO precursor layer (Table S1) was mounted on the six-axis Stewart platform, and the Ag–rGO precursor layer was ablated by focusing a femtosecond laser on the substrate surface. The substrate was then washed with DI water several times until the entire laser-untreated area was cleaned thoroughly. The residual pattern was dried under ambient conditions.

The silica substrate coated with the GO layer was first fixed at the bottom of a flat-bottomed container. Subsequently, a mixture of silver nitrate and sodium citrate was poured into the container to ensure that the substrate was completely immersed in the liquid. To obtain the hybrid structure, the laser was focused on the interface between the liquid and the GO layer. After the writing, the substrate was washed with DI water several times until the entire laser-untreated area was cleaned thoroughly. The residual pattern was dried under ambient conditions. The details of the parameters of the processing environment are presented in Table S1.

Table S1. Main Parameters of the Different Synthesis Methods.

Number	Processing environment	Substrate	Layer component of the precursor	Objective	Scanning speed
FigureS2 (a)	Air	Si	$AgNO_3$-GO mixed layer	50X NA=0.5	200um/s
Figure S2 (b)	Air	SiO_2	$AgNO_3$-GO mixed layer	50X NA=0.5	200um/s
Figure S2 (c)	liquid	SiO2	Go Layer	100mm plano-convex lens	500um/s

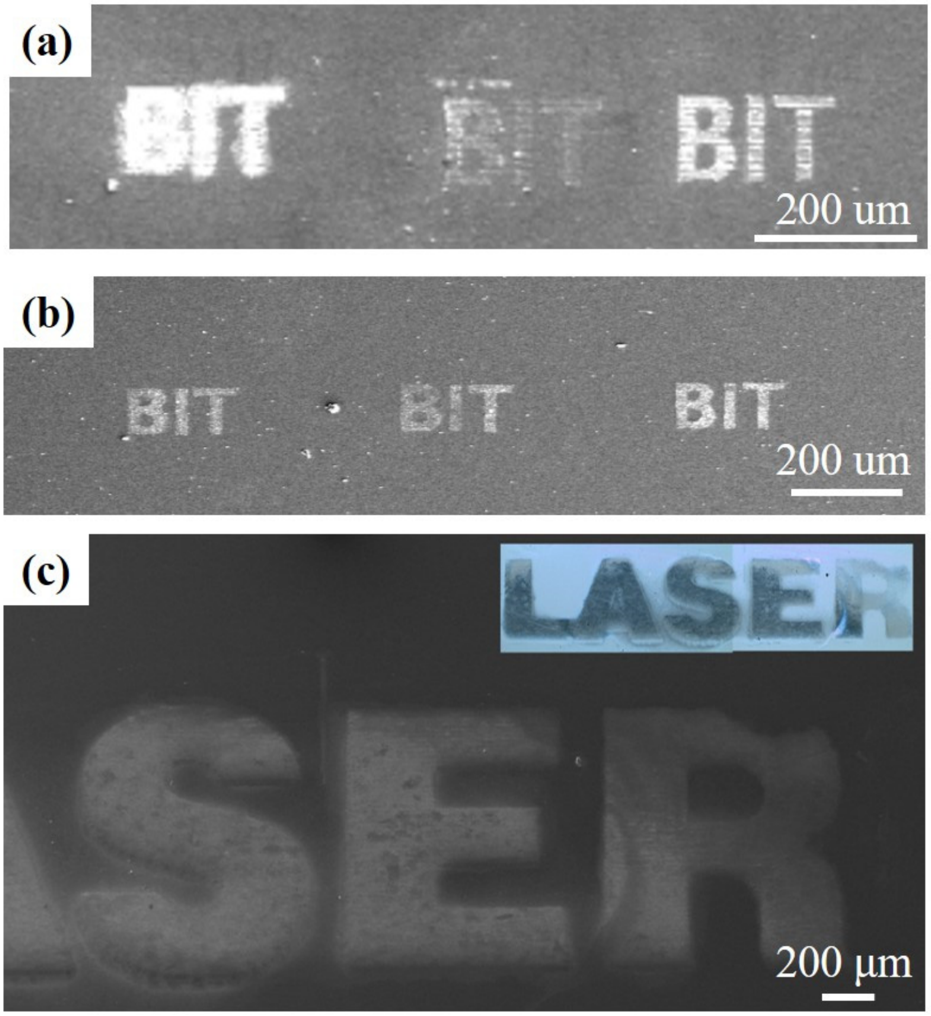

Figure S4. Mapping of the Ag–rGO hybrid under different conditions/with various substrates: (a) direct writing in air on the Si substrate, (b) direct writing in air on the SiO_2 substrate, and (c) direct writing under Ag–rGO on the SiO_2 substrate under liquid conditions.

5. Morphology of the GO without Ag doping

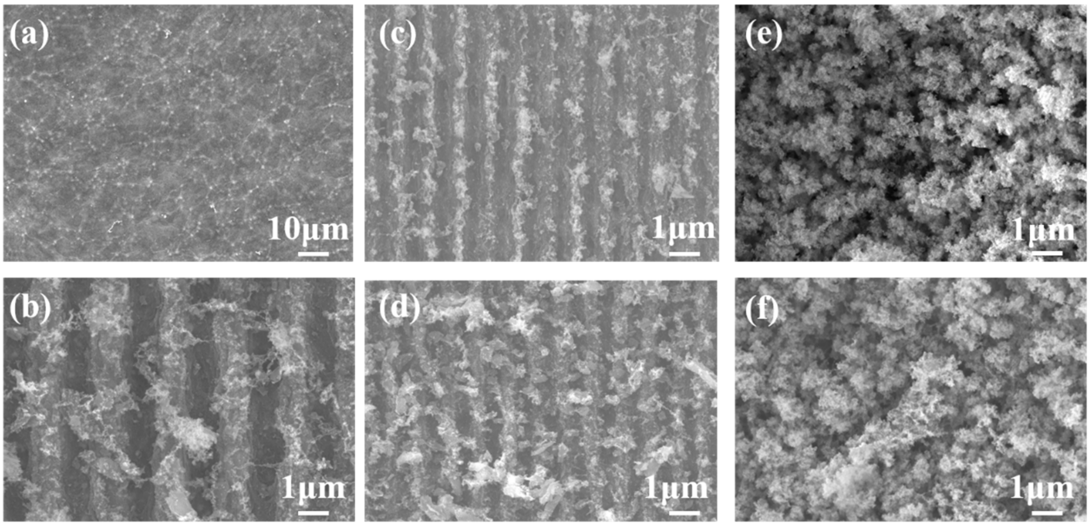

Figure 1. Micromorphology of the GO on SiO_2 treated with a femto-second laser at the following fluences: (a) Before laser treatment, (b) 0.035 J/cm^2, (c) 0.071 J/ cm^2, (d) 0.354 J/ cm^2, (e) 0.708 J/ cm^2, and (f) 1.288 J/cm^2.

Compared with the micrographs shown in Figure 1, coral-loid-like structure and nanoparticles which are important for inducing the local plasmon resonance are not observed as shown in Figure R1 (b)-(d). When the fluenc increase to the 0.708 J/ cm^2, GO sheet cluster together as shown in Figure R1 (e) and (f). Thus, the reduction of silver can change the morphology of Ag- rGO hybrid and promote the melting of the recast layer

Reference

1. Zhu, C.; Guo, S.; Fang, Y.; Dong, S., Reducing sugar: new functional molecules for the green synthesis of graphene nanosheets. *ACS nano* **2010,** 4, (4), 2429-2437.
2. Alazmi, A.; Rasul, S.; Patole, S. P.; Costa, P. M., Comparative study of synthesis and reduction methods for graphene oxide. *Polyhedron* **2016,** 116, 153-161.
